# Plantar pressures values related with appearance of mechanical hyperkeratosis before and after surgery of mild hallux valgus

**DOI:** 10.3389/fmed.2023.1141091

**Published:** 2023-04-12

**Authors:** Alfonso Martínez-Nova, Jaime Gascó-López de Lacalle, Juan Francisco Morán-Cortés, Juan Diego Pedrera-Zamorano, Raquel Sánchez-Rodríguez

**Affiliations:** ^1^Department of Nursing, University of Extremadura, Plasencia, Spain; ^2^School of Health Sciences, Catholic University of Valencia San Vicente Mártir, Valencia, Spain

**Keywords:** hyperkeratoses, plantar pressure, BioFoot, surgery, forefoot

## Abstract

**Background:**

Hyperkeratoses are thickenings of the stratum corneum, provoked by deviation of the ray and excessive plantar pressures. They are very common under the first metatarsal head (MTH) and on the big toe when there exists hallux valgus. The objective of this study was to assess plantar pressures pre- and post-surgery to try to define the threshold values that could determine the appearance of keratopathies.

**Materials and methods:**

Seventy-nine patients (100 feet) who had undergone percutaneous distal soft-tissue release and the Akin procedure were evaluated prospectively. The BioFoot/IBV^®^ in-shoe system was used for objective baropodometric functional evaluations of the heel, midfoot, first through fifth MTHs, hallux, and lesser toes. The presence or absence of a hyperkeratosis (HK) or plantar callus under the first MTH or hallux was recorded. The average follow-up time at which the measurements were repeated was 28.1 months.

**Results:**

Pre-surgery, 62 feet presented a painful HK on the big toe, while post-surgery, only 9 of the feet presented the same lesion. Patients who presented a prior HK at the first metatarsophalangeal (MTP) joint had a mean pressure of 417.2 ± 254.5 kPa as against a value of 359.6 ± 185.1 kPa for the rest. Post-surgery, these values dropped to 409.8 and 346.3 kPa, respectively.

**Conclusion:**

Patients with HK presented an 11% greater mean pressure than those without. The values obtained with the BioFoot/IBV^®^ system in the present study can therefore be considered predictive of the appearance of HK under the first MTH and on the side of the big toe.

## 1. Introduction

Hyperkeratosis (HK) is a thickening of the stratum corneum provoked by excessive proliferation of corneocytes. It is a physiological mechanism of the skin to counteract excessive plantar pressure or friction between the surface of the skin and footwear (1). It becomes pathological, however, when the concentration of keratin exceeds normal limits and causes pain. It is a frequent phenomenon, affecting 20% of the general population, it occurs more often in women and the elderly, and is becoming one of the commonest lesions seen in podiatry consultations (1). Despite being a frequent phenomenon, it is still unknown what the key pressure threshold is determining the appearance of this keratopathy (2).

One of the main causes of the appearance of HK is the presence of a pathology such as hallux valgus (HV) (3). In HV, there are altered plantar loading patterns, with pathological increases under the first, second, and third metatarsal heads (4, 5) relative to healthy subjects. These loading alterations are usually associated with the appearance of painful HK under the first metatarsal head (MTH) or on the side of the big toe (interphalangeal joint, IPJ).

Hallux valgus surgery with correct alignment of the foot deformity may be an important factor for potentially reducing the plantar HK pattern (6–8). If the HK under the first MTH is considerably reduced or disappears after HV surgery, this could be because the surgical correction of the deformity lowered the plantar pressures (4, 9, 10). Nonetheless, the threshold pressures for either the appearance of a keratopathy or its disappearance after surgical correction remain unknown.

**FIGURE 1:**
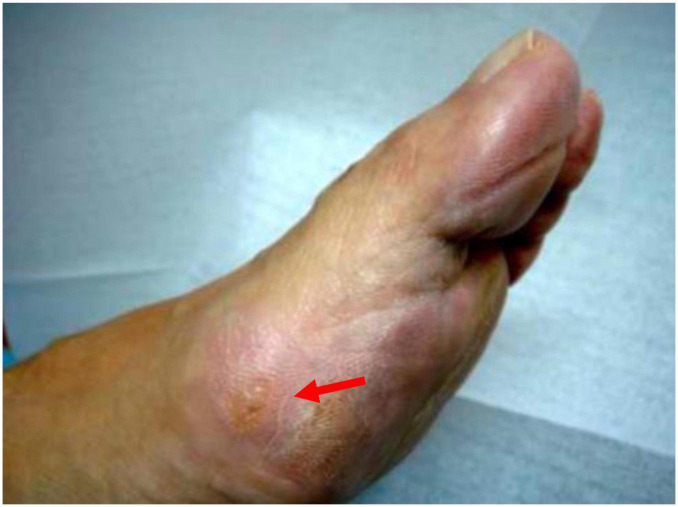
Hyperkeratosis in 1st metatarsophalangeal (MTP) joint.

**FIGURE 2:**
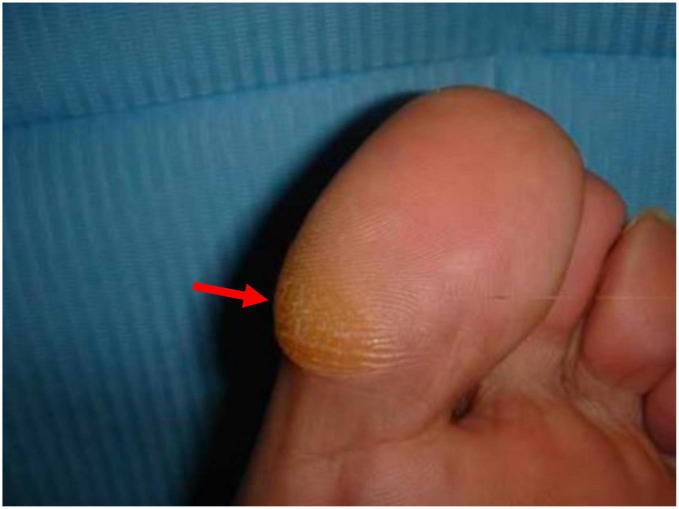
Hyperkeratosis in interphalangeal joint (IPJ).

The objective of this study was based on the hypothesis that, in feet with mild HV, hyperpressures would be found in the area of the first ray’s metatarsal segment (Figure 1) which could lead to the associated HK in the medial zone of the first metatarsophalangeal (MTP) joint and of the big toe (Figure 2). Likewise, the study is based on the hypothesis that the percutaneous distal soft-tissue release (DSTR) and AKIN surgical technique would modify the plantar pressure pattern in the forefoot, bringing the plantar values closer to those established as normal, and that therefore the pattern of plantar HK would disappear. Therefore, the objective of the study was to assess the pre- and post-surgical plantar pressures in the area of the first ray and compare them so as to try to define the threshold values that could determine the appearance of keratopathies.

## 2. Materials and methods

### 2.1. Patients

The sample consisted of 100 cases of mild HV treated using the percutaneous DSTR-AKIN technique, corresponding to 79 patients (21 with bilateral interventions and 58 treated unilaterally). All the patients were women. They met the requirements and agreed voluntarily to be part of the study. Their mean age was 54.7 ± 12.5 years (range, 27–81), mean weight 65.9 ± 9.4 kg (range, 45–86), mean height 161.9 ± 5.5 cm (range, 150–179), and BMI 25.1 ± 3.2 kg/m^2^ (range, 15.5–33). After proposing their participation in the study, the patients were given an informative document that explained the nature of the research and its objectives. After reading it and having any possible doubts clarified, they gave their informed consent to participate. This study was conducted in accordance with recommendations of the latest version of the Declaration of Helsinki. The Ethics Committee of the University of Extremadura (file n° 102/2007) gave the research project a positive report, approving the study procedures. Of the 100 operations performed, 53 (53%) were on right feet and 47 (47%) on left feet.

### 2.2. Inclusion and exclusion criteria

The inclusion criteria for applying the percutaneous DSTR-AKIN procedure were: mild or painful HV (11–14) with first intermetatarsal angle (IMA) ≤13° (15) and hallux abductus angle (HAA) in the range 15°< HAA ≤ 30° (15) presenting positional deformity, a normal forefoot adduction angle < 14°, and no arthrosis or osteoporosis. The general inclusion criteria for the study were: patients who participated voluntarily giving their signed consent, with all functional, radiological, and baropodometric data available and error free, and who were available for 1 year of follow-up. The general exclusion criteria of the study were: patients with moderate or severe HV, having previously undergone foot surgery, or having presented serious lower-limb lesions or alterations during the preceding 12 months. There was a previous medical evaluation, to rule out problems with the surgery, and thus ensure that the patients included did not present co-morbidities, such as diabetes, venous insufficiencies, etc., that could contraindicate surgery.

### 2.3. Registering HK and plantar pressures: First measurement

**FIGURE 3:**
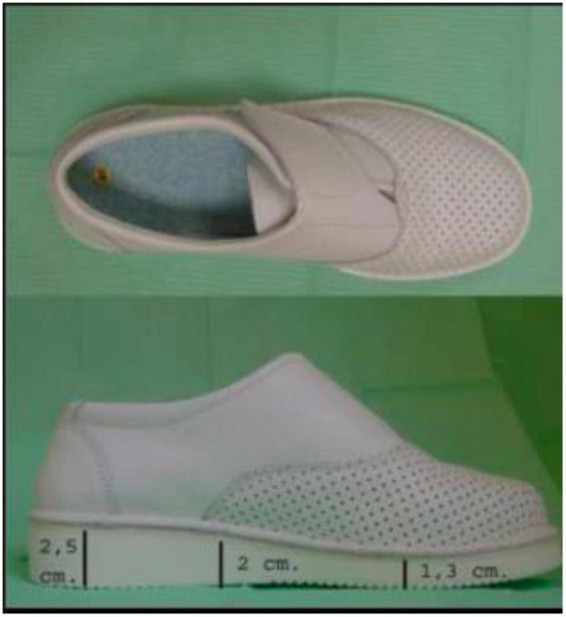
Footwear used in study.

**FIGURE 4:**
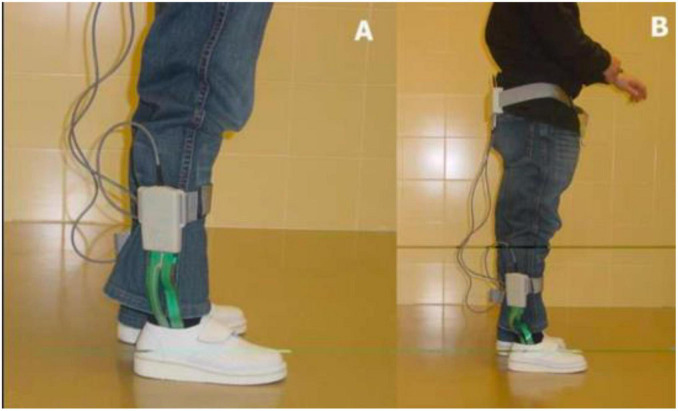
**(A)** Participant with instrumented insoles. **(B)** Participant with the complete BioFoot/IBV^®^ system equipment ready for the measurement.

**FIGURE 5:**
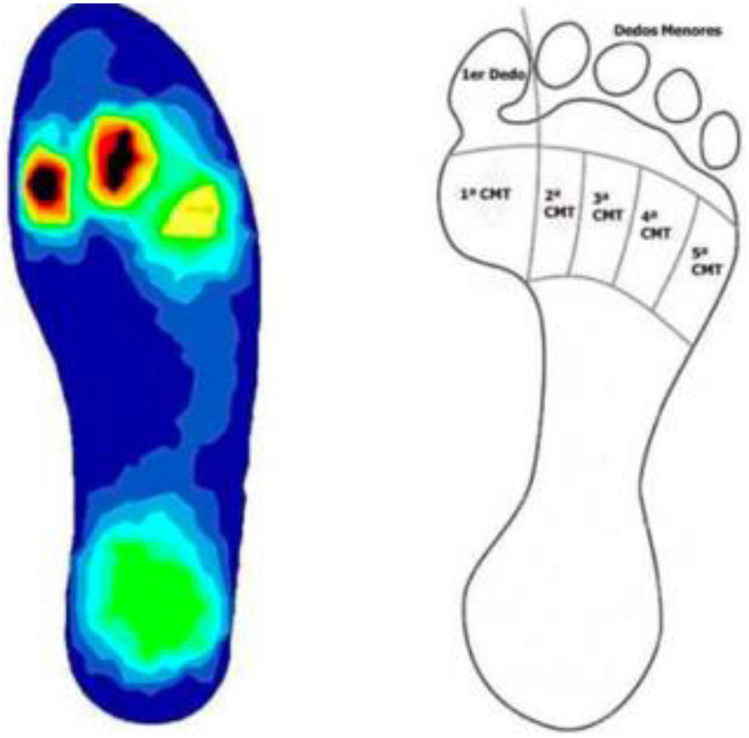
Forefoot division in the seven zones analyzed.

The presence or absence of HK in the areas of the first MTH and the ball of the big toe were noted. To avoid differences in the measurements caused by each subject’s personal footwear, they were all measured with the same model of footwear (closed clog, ZALE^®^, Alicante, Spain) (Figure 3). The baropodometric analysis was performed with the BioFoot/IBV^®^ system of instrumented insoles (Figure 4). After accommodating the patient to the system, the parameters were set to 100 Hz, and 3 measurements of 8 s were taken, allowing the analysis of from 12 to 15 steps. The mean pressure in the plantar area of the first MTH and the big toe was analyzed (Figure 5).

### 2.4. Surgical technique

**FIGURE 6:**
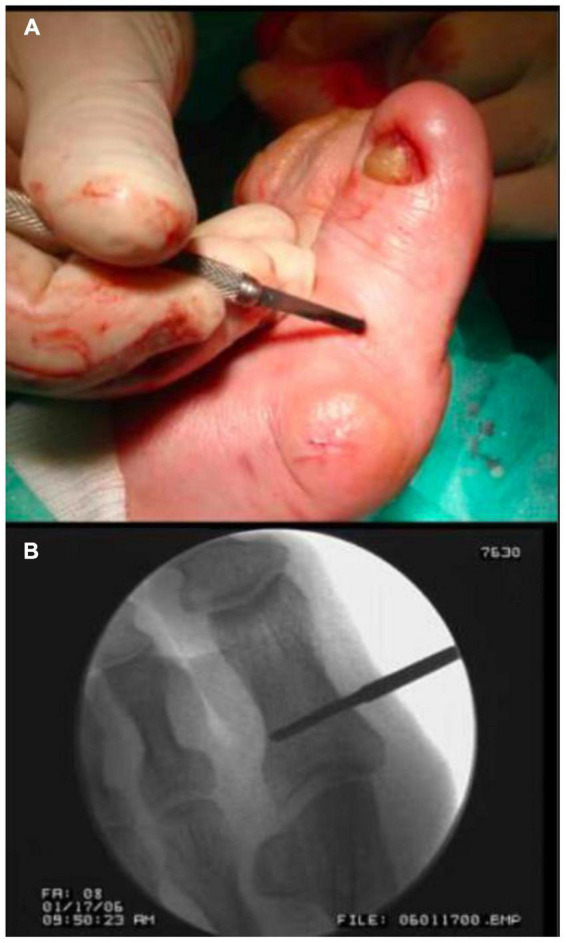
Application of the surgical technique. Tenotomy of the big toe abductor tendon **(A)** and Akin’s osteotomy **(B)**.

**FIGURE 7:**
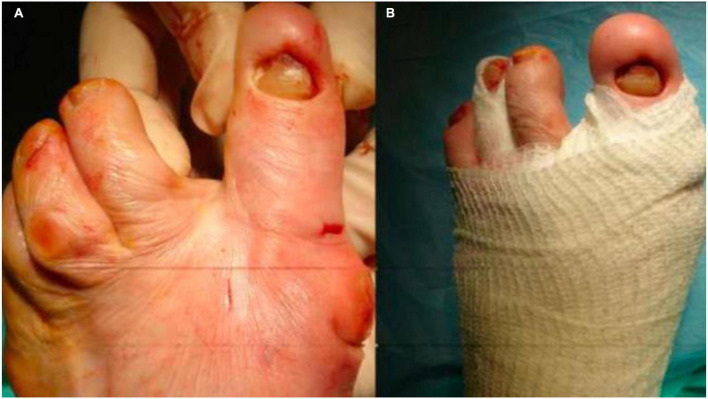
Alignment **(A)** and bandaging of the first digit-metatarsal segment **(B)**.

The complete surgical technique was performed under total anesthetic block of the foot. It comprised the following procedures: exostectomy, tenotomy of the big toe abductor tendon, lateral capsulotomy, and Akin osteotomy (Figures 6, 7). This technique has been shown to be very effective in the treatment of mild HV (15, 16).

### 2.5. Second measurement

The presence or absence of HK and the plantar pressure measurements were performed again after an average follow-up period of 2 years (mean 28.1 months, range 24–33 months).

### 2.6. Statistical analysis

Descriptive analysis (Table 1) of the pre-and post-surgery baropodometric variables (mean ± standard deviation). Table of frequencies and χ^2^ test (Table 2) between the presence and absence of HK. Application of k-means clustering to the mean first MTH pressure and keratopathy on the side of the first MTP joint and the mean big toe pressure and keratopathy on the side of the IPJ (Table 3). Calculations were done using the SPSS v.15.0 software package (SPSS, Chicago, IL) for Windows (UEX campus license). The significance level was set at 5% (*p* < 0.05).

**TABLE 1:** Modification of pre and post-surgical mean pressure values.

Zone	Mean ± SD	Mean ± SD	*p*-value
***n* = 100**			
	Before surgery	After surgery	
1st MTH	375.8 ± 207.2	360.1 ± 207.7	0.552
2nd MTH	421.4 ± 287.6	412.5 ± 200.1	0.327
3rd MTH	380.6 ± 263.8	390.7 ± 213.4	0.727
4th MTH	251.5 ± 195.9	322.2 ± 215.1	0.002*
5th MTH	139.1 ± 119.3	176.3 ± 132.7	<0.001*
1st finger	328.5 ± 113.2	151.9 ± 37.9	<0.001*
Minor fingers	96.6 ± 69.1	122.9 ± 81.6	0.004*

According to student’s *t*-test for paired samples. *p < 0.05 (SD, standard deviation; MTH, metatarsal head).

**TABLE 2:** Hyperkeratosis: pre-and post-surgery results and χ^2^ test.

(*n* = 100)		Pre-surgery	Post-surgery	*p*-value
	No	10	80	
Hyperkeratosis	1st MTP joint	28	11	<0.01*
	Big toe IPJ	62	9	

*p < 0.05.

**TABLE 3:** Mean pressure under the first MTH and big toe, k-means clustering.

HK 1st MTP joint		Yes	No
		***n* = 28**	***n* = 72**
Pressure 1st MTH	Pre	417.2 ± 254.5	359.6 ± 185.1
	Post	409.8 ± 280.4	346.3 ± 124.3
**HK big toe IPJ**		**Yes**	**No**
		***n* = 62**	***n* = 38**
Pressure big toe	Pre	369.5 ± 108.7	261.6 ± 86.1
	Post	216.1 ± 25.3	156.7 ± 20.7

HK, hyperkeratosis; MTH, metatarsal head; Pre, pre-surgery; Post, post-surgery; IPJ, interphalangeal joint.

## 3. Results

Table 1 shows the descriptive analysis of the pre-and post-surgery baropodometric variables with mean and standard deviation.

Pre-surgery, 62 feet presented a painful HK under the big toe, while only 9 of the feet presented the same lesion post-surgery (Table 2). This difference was statistically significant (*p* < 0.01).

The k-means cluster analysis indicated that the patients presenting pre-surgery HK in the medial area of the first MTP joint had a mean pressure of 417.2 ± 254.5 kPa vs. 359.6 ± 185.1 kPa for the rest. Post-surgery, these values dropped to 409.8 and 346.3 kPa, respectively (Table 3). Post-surgery, all the HK were superficial, with no single case of deeper HK that could lead to a possible ulceration.

In relation to the pressure under the big toe, patients presenting HK under the IPJ had a pressure of 369.5 ± 108.7 kPa vs. 261.6 ± 86.1 kPa for the rest. Post-surgery, these values dropped to 216.1 and 156.7 kPa, respectively (Table 3).

## 4. Discussion

The k-means clustering analysis provided interesting data regarding the etiology of the appearance of keratopathies in the medial zone of the first MTH and the big toe. The patients who presented HK under the first MTP joint had a pressure of 417 kPa, while those who presented it under the big toe had a pressure of 369.5 kPa (Table 2). These values are above the 200 kPa for the forefoot region established as “normal” for the same BioFoot/IBV^®^ system (17–19).

Hyperpressures occurring on the sole of the foot are one of the commonest causes of pain and discomfort associated with walking and the use of footwear. Despite this fact, there have been few studies on the effect that certain levels of pressure have on the feeling of comfort of the foot. No studies have identified the threshold values beyond which pain or keratopathies can develop (3). Only Waldecker (20) defined values above 700 kPa as possibly being indicative of metatarsalgia, but there are no reliable data with the BioFoot/IBV^®^ system.

Hyperkeratoses are mechanically induced lesions, and, although they are one of the commonest podiatric problems and occur in a high percentage of the population, especially in older people (21, 22), their etiology is still poorly known (23, 24). Treatment of plantar HK includes the scalpel removal and also, keratolytic topical treatments to reduce the HK thickness, like high concentration urea (40%) in occlusion and patches of lactic or salicylic acids (25).

The development of HK is a response to repetitive pressure or friction in a certain area. The skin then undergoes accelerated keratinization and a lower rate of desquamation, leading to increased thickness of the stratum corneum (26). This is a normal protection mechanism of the skin, preventing deep tissue damage by spreading applied forces over a greater area and volume of skin (27). High plantar pressures may play an important role in the development of HK and accelerate the process of keratinocyte formation.

When this process advances with greater friction or pressure, the HK is accentuated, especially in thickness, and can act as a foreign body, causing discomfort and pain (26). Furthermore, in diabetics, these high pressures can damage deeper tissues and lead to ulceration (28, 29).

Hyperkeratoses are usually treated by scalpel delamination to relieve the pain. Callus pads, silicone orthoses, or plantar supports are used to avoid their reappearance (30, 31).

Although high plantar pressures have commonly been associated with HK formation, only two research studies have provided evidence that plantar HKs develop in areas of high pressure, this being especially the case in older people (23, 32). Although Pooter and Pooter (32) found that pressures in people with HK were up to 25% greater than in a control group, their study did not control well some factors that could influence plantar pressures, such as gait rate (26), weight (33), or associated deformities (12, 17), and, despite there being a belief that the removal of HK reduces the underlying plantar pressure (31), those workers found no difference in peak plantar pressures after the removal of HK. Also, Menz and Zammit (23) found that people with HK on the big toe had a 12.3% greater pressure value than a control group without HK.

In a baropodometric study about surgery for mild HV, Cancilleri et al. (34) found that the surgical technique with Boc’s modification reduced the incidence of HK and helomas under the second and third MTHs. This low incidence of HK was accompanied by a reduction of pressure in those areas.

Obtaining the threshold value for the appearance of HK is important, since baropodometric screening of patients with mild HV could indicate when they might develop a painful keratopathy, and hence preventive treatment could be initiated (35). In the present series of patients, the pre- and post-surgery values obtained suggest that the threshold value should be close to 180 kPa for those subjects who have undergone surgery for mild HV and close to 300 kPa for those subjects who have not.

## 5. Conclusion

The patients with HK in the present study had an 11% greater mean pressure than those without HK. Thus, the values obtained with the BioFoot/IBV^®^ system can be taken as predictive of the appearance of HK under the first MTH and on the side of the big toe. In exploratory baropodometric analyses, elevated plantar pressures could serve as a guide toward initiating preventive treatments, even when HK has yet to appear. This sign could also be very useful for the prevention of ulcers in diabetics.

## Data availability statement

The raw data supporting the conclusions of this article will be made available by the authors, without undue reservation.

## Ethics statement

The studies involving human participants were reviewed and approved by the Ethics Committee of the University of Extremadura (file n° 102/2007). The patients/participants provided their written informed consent to participate in this study.

## Author contributions

AM-N participated in the sampling with the subjects and statistical analysis. JG-L participated in writing the manuscript. JM-C and RS-R participated in the statistical analysis. JP-Z participated in the revision of the manuscript. All authors contributed to the article and approved the submitted version.
